# Emergency management: orbital cellulitis

**Published:** 2018-11-09

**Authors:** Fatima Kyari

**Affiliations:** 1 Consultant Ophthalmologist Medical Education Coordinator, Baze University, Abuja, Nigeria.

**Orbital cellulitis is an infection of the deep tissues of the orbit. It is life-threatening, as infection can easily spread into the brain.**

**Figure:**
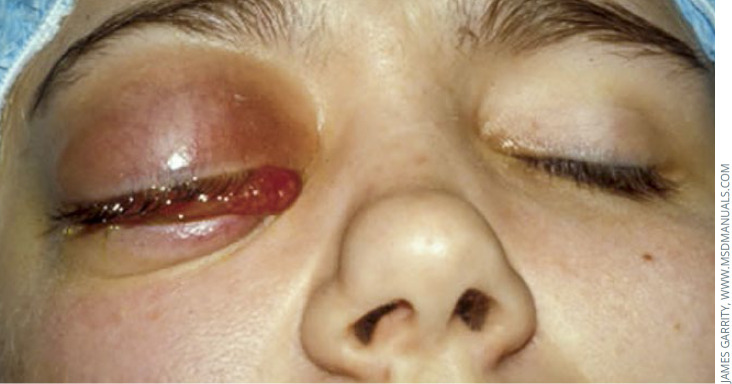
Orbital cellulitis. Clinical signs include proptosis, peri-orbital skin erythema, lid oedema and conjunctival chemosis.

Orbital cellulitis is usually the result of infection that has originated in the paranasal sinuses and spread to the orbit. It may arise due to the spread of minor infections of the eyelid, the face and the lacrimal sac or, rarely, from endophthalmitis. Orbital cellulitis can also occur when infections in other parts of the body spread through the bloodstream (haematogenous spread). The organisms most commonly isolated from orbital infections are *Staphylococcus spp., Streptococcus spp.* and *Haemophilus spp*.

If treatment is inadequate and/or delayed, vision loss, cavernous sinus thrombosis, intracranial abscess, meningitis, osteomyelitis and even death can occur within a short time.

Orbital cellulitis is an emergency and admission and in-patient management must be instituted immediately.

## Symptoms

Patients may present with pain, fever (especially in children), frontal headache, swelling of the eye or double vision. There is often a history of upper respiratory tract infection.

## Signs

Clinical signs include:

- Proptosis (forward displacement of the eye)
- Peri-orbital skin erythema (redness) and lid oedema
- Conjunctival chemosis (inflammation and swelling of the conjunctiva).
- Limited eye movement.

Due to the proptosis, there may be exposure keratopathy with corneal ulceration.

The optic nerve head may be swollen.

Visual acuity may be decreased. Vision loss may be due to corneal ulceration or ischaemic necrosis of the optic nerve due to mechanical pressure. However, vision loss is often temporary and improves with treatment.

The patient is usually unwell, as there may be associated sepsis, with fever, nausea and vomiting, and even cognitive impairment and confusion.

## Remedies and immediate management

1. Immediately begin systemic (intravenous) antibiotic treatment using broad-spectrum antibiotics that are effective against most Gram positive and Gram negative bacteria. A combination of **third-generation cephalosporin** and **flucloxacillin** is recommended.
2. Make the patient comfortable: relieve pain, treat fever and prevent/treat vomiting and dehydration.
3. Investigate the primary source of infection if possible.

The single most important imaging investigation in orbital cellulitis with suspected orbital abscess is a CT scan, as this would aid in the detection and demarcation of the abscess and assessment of the sinuses. However, this may not be possible in resource-poor environments. Ocular ultrasonography is also useful in cases of orbital abscess, and plain sinus X-rays may show air-fluid level in the sinuses.

## Referral and/or follow-up

Surgical drainage of orbital or subperiosteal abscess is indicated in some cases of orbital cellulitis. These patients should be referred urgently for surgery as delayed intervention is associated with poor results.

Careful monitoring of patients is essential, as they may need oral antibiotics after the initial intravenous antibiotics. Children with vision loss should be followed up to measure their visual acuity and assess whether they need further treatment.

## Be prepared

- Ensure that everyone with eye care responsibilities knows the signs of orbital cellulitis: it is an emergency that can result in blindness and death
- Create a poster or protocol that lists the clinical signs and what to do. You can download the images in this issue from **www.flickr.com/photos/communityeyehealth/**
- Put together an orbital cellulitis pack containing an IV bag, antibiotics and a set of instructions for preparing the antibiotics. Check regularly to ensure that the medicines are in date
- Practise preparing the IV bag and antibiotics.
